# Molecular Confirmation of Autochthonous *Taenia saginata* Infection, Timor-Leste, 2019

**DOI:** 10.3201/eid3207.252034

**Published:** 2026-07

**Authors:** Hanna Jin, Sung-Tae Hong, Merita Antonio Armindo Monteiro, Endang da Silva, Odete da Silva Viegas, Felix dos Santos Lopes, Dong Hee Kim, Sung Hye Kim

**Affiliations:** Seoul National University College of Medicine, Jongno-gu, South Korea (H. Jin, S.-H. Hong, D.H. Kim); National Institute of Public Health, Dili, Timor-Leste (M.A. Armindo Monteiro, E. da Silva); Ministry of Health, Dili (O. da Silva Viegas); World Health Organization, Timor-Leste Country Office, Dili (F. dos Santos Lopes); Hanyang University College of Medicine, Seongdong-gu, South Korea (S.H. Kim)

**Keywords:** parasites, human taeniasis, *Taenia saginata*, beef tapeworm, COX1 gene, One Health, Timor-Leste

## Abstract

We report a case of autochthonous *Taenia saginata* infection in Timor-Leste. Screening of 1,121 schoolchildren revealed a 0.4% prevalence of human taeniasis. Genetic analysis of the mitochondrial *cox1* gene identified group A lineage. Our findings fill a considerable geographic data gap and highlight the need for integrated One Health control strategies.

Human taeniasis is a foodborne neglected tropical disease caused by 3 species of tapeworms: *Taenia solium*, *Taenia saginata*, and *Taenia asiatica*. Although the parasites are endemic across Africa and the Americas, Asia is unique for the sympatric distribution of all 3 species, particularly in rural areas where traditional livestock rearing persists (*1*). Among these species, the *T. saginata* beef tapeworm is the most common zoonotic tapeworm globally; infection occurs through the consumption of raw or undercooked beef containing cysticerci (*2*). The epidemiologic landscape in Southeast Asia is complex because of the genetic relationship between *T. saginata* and *T. asiatica* tapeworms (*3*). Recent molecular analyses have revealed that the 2 are sister species that are not completely reproductively isolated; consequently, many adult worms circulating in the region, including those in Indonesia, are hybrid-derived descendants (*4*).

Timor-Leste, a Southeast Asia nation sharing the island of Timor with Indonesia, has long represented a considerable geographic data gap. A 2020 systematic review covering 1990–2017 found no retrievable data for the country (*2*), leaving its endemic status unconfirmed, despite cultural practices favoring beef consumption and a population of ≈225,000 cattle (*5*).

In early 2019, as part of a national monitoring program for soil-transmitted helminthiasis, we collected fecal samples from 1,121 schoolchildren across 6 schools in Timor-Leste. Initial screening using the Kato-Katz thick-smear technique identified 4 children as *Taenia* spp. egg–positive, representing an overall prevalence of 0.4%, which is consistent with regional pediatric data from countries such as Myanmar (*6*). After the identification of eggs, we administered a single oral dose of praziquantel (10 mg/kg) to affected children, followed by a purge with a mild laxative solution (Colonlyte powder; Dream Pharma [now Alvogen Korea], https://www.alvogenkorea.com) so that we could recover intact tapeworm segments. We successfully recovered intact proglottids from an asymptomatic 11-year-old girl residing in the capital, Dili. Of note, the patient had no history of international travel, confirming that the infection was autochthonous.

The recovered tapeworm segments were flat and creamy-white and measured 12–15 mm in length (Figure 1). Acetocarmine staining revealed 18–20 lateral uterine branches and a lack of rostellar hooks on the scolex, which served to morphologically exclude the *T. solium* pork tapeworm. To achieve definitive species identification, we extracted genomic DNA using the DNeasy Blood & Tissue Kit (QIAGEN, https://www.qiagen.com) and targeted a fragment of the mitochondrial *cox1* gene for PCR amplification using T1F and T1R primers as described previously (*7*). Sequencing of the amplicons showed a 99.58% identity with *T. saginata* (GenBank AB984348.1) and a marked genetic divergence from *T. solium*. Phylogenetic analysis using the neighbor-joining method placed the Timor-Leste isolate firmly within the group A clade, distinct from *T. asiatica* and *T. solium* lineages (Figure 2; Appendix). The high bootstrap support (>90%) for those nodes reinforces the isolate’s genetic alignment with regional *T. saginata* populations rather than the hybrid-derived descendants of *T. asiatica* and *T. saginata* tapeworms found in nearby North Sumatra (Figure 2).

**Figure 1:**
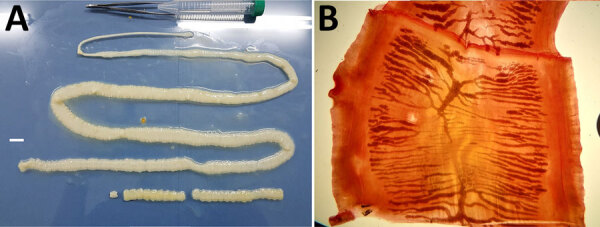
Proglottids of *Taenia saginata* beef tapeworm collected from an 11-year-old girl in Dili, Timor-Leste, informing an investigation focused on molecular confirmation of autochthonous *T. saginata* infection, Timor-Leste, 2019. A) Gross morphology of proglottids, showing flat, creamy-white segments, measuring 12–15 mm in length. The scolex was confirmed to lack rostellar hooks on gross examination. Scale bar = 1 cm. B) Acetocarmine-stained proglottid showing 18–20 lateral uterine branches, morphologically consistent with *T. saginata* tapeworm and excluding *T. solium* tapeworm.

**Figure 2:**
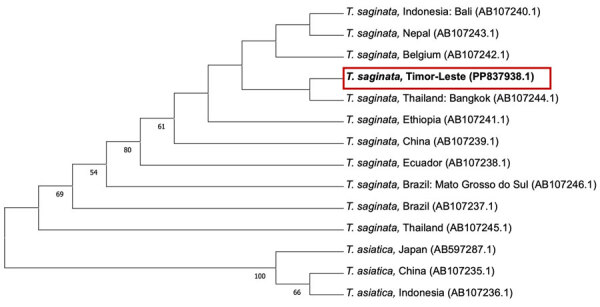
Phylogenetic analysis of the *cox1* gene from a child with taeniasis in a study revealing molecular confirmation of autochthonous *Taenia saginata* infection, Timor-Leste, 2019. Phylogenetic tree of *T*. *saginata* and related taxa was based on partial mitochondrial *cox1* gene sequences (458 bp). The evolutionary history was inferred by the neighbor-joining method with 1,000 bootstrap replicates; bootstrap values (>50%) are shown at branch nodes. Evolutionary distances computed using Kimura 2-parameter model. Analyses performed in MEGA 11 (https://www.megasoftware.net). The Timor-Leste isolate (GenBank PP837938.1, red box) clusters within the group A clade of *T. saginata*, distinct from *T. asiatica* (outgroup) and *T. solium* lineages. GenBank accession numbers are shown in parentheses.

Combined with the emergence of a concurrent *T. solium* infection that was molecularly confirmed in a 10-year-old child in Dili during the same surveillance period (*7*), our results confirm the co-endemicity of *T. saginata* and *T. solium* tapeworms in Timor-Leste. Although the *T. saginata* isolate is linked to regional Southeast Asian genotypes (group A), the *T. solium* case showed homology with Madagascar lineages, suggesting different historical introduction routes for the 2 species (*7*). The detection of the parasites in schoolchildren suggests transmission risks within the home environment and the early cultural integration of beef consumption (*1*,*8*).

The reliance on Kato-Katz smears in regional surveillance remains a hurdle, because this analysis cannot differentiate between *Taenia* species. Furthermore, the documented presence of hybrid-derived descendants in neighboring Indonesia (*4*) suggests that future surveillance should incorporate nuclear markers to complement mitochondrial genotyping. Such a dual-marker approach is essential to detect potential interspecific hybridization or introgression that may be masked by mitochondrial analysis alone. Nevertheless, the confirmation of endemicity for both *T. saginata* and *T. solium* tapeworms warrants enhanced integrated surveillance to accurately identify co-circulating *Taenia* species. The concurrent detection of *T. solium* tapeworm in this pediatric cohort underscores the need for species-discriminating molecular diagnostics and cross-border cooperation to prevent neurocysticercosis, the public health consequences of which far exceed those of *T. saginata* taeniasis (*9*).

Appendix Additional information for molecular confirmation of autochthonous *Taenia saginata* infection, Timor-Leste, 2019.

## References

[1] Ito A, Li T, Wandra T, Dekumyoy P, Yanagida T, Okamoto M, et al. Taeniasis and cysticercosis in Asia: a review with emphasis on molecular approaches and local lifestyles. Acta Trop. 2019;198:105075. 10.1016/j.actatropica.2019.105075 31295430

[2] Eichenberger RM, Thomas LF, Gabriël S, Bobić B, Devleesschauwer B, Robertson LJ, et al. Epidemiology of *Taenia saginata* taeniosis/cysticercosis: a systematic review of the distribution in East, Southeast and South Asia. Parasit Vectors. 2020;13:234. 10.1186/s13071-020-04095-1 32381027 PMC7206752

[3] Jeon HK, Eom KS. Taenia asiatica and Taenia saginata: genetic divergence estimated from their mitochondrial genomes. Exp Parasitol. 2006;113:58–61. 10.1016/j.exppara.2005.11.018 16546174

[4] Zein U, Siregar S, Janis I, Pane AH, Purba JM, Sardjono TW, et al. Identification of a previously unidentified endemic region for taeniasis in North Sumatra, Indonesia. Acta Trop. 2019;189:114–6. 10.1016/j.actatropica.2018.10.004 30321521

[5] Ministry of Agriculture and Fisheries. Timor-Leste Agriculture Census 2019 National Report. 2023 [cited 2025 Dec 25]. https://inetl-ip.gov.tl/2023/03/16/2241/

[6] Won EJ, Jung B-K, Song H, Kim M-S, Kim H-S, Lee KH, et al. Molecular diagnosis of *Taenia saginata* tapeworm infection in 2 schoolchildren, Myanmar. Emerg Infect Dis. 2018;24:1156–8. 10.3201/eid2406.180217 29774855 PMC6004857

[7] Jin H, Hong S-T, Monteiro MAA, da Silva E, da Silva Viegas O, Dos Santos Lopes F, et al. Molecular confirmation of Taenia solium taeniasis in child, Timor-Leste. Emerg Infect Dis. 2024;30:1964–7. 10.3201/eid3009.240238 39174025 PMC11346990

[8] Wandra T, Ito A, Swastika K, Dharmawan NS, Sako Y, Okamoto M. Taeniases and cysticercosis in Indonesia: past and present situations. Parasitology. 2013;140:1608–16. 10.1017/S0031182013000863 23965293

[9] Food and Agriculture Organization of the United Nations. Transforming the livestock sector through the Sustainable Development Goals. Rome, 2018. Licence: CC BY-NC-SA 3.0 IGO [cited 2025 Dec 25]. https://openknowledge.fao.org/server/api/core/bitstreams/d67a99fb-66ca-42a0-b5ac-3f907b35f09f/content

