# Supplementary material for: Molecular Confirmation of Autochthonous Taenia saginata Infection, Timor-Leste, 2019
Source: Emerg Infect Dis. 2026 Jul;32(7):1204–6. doi: 10.3201/eid3207.252034 (PMC13322410; doi:10.3201/eid3207.252034)
Supplement: Appendix — Additional information for molecular confirmation of autochthonous Taenia saginata infection, Timor-Leste, 2019. [file 25-2034-Techapp-s1.pdf]

Article DOI: http://doi.org/10.3201/eid3207.252034

EID cannot ensure accessibility for supplementary materials supplied by authors. Readers who have difficulty accessing supplementary content should contact the authors for assistance.

Molecular Confirmation of Autochthonous *Taenia saginata* Infection, Timor-Leste, 2019

Appendix

Evolutionary relationships of taxa

The evolutionary history was inferred using the Neighbor-Joining method (*1*). The optimal tree is shown. The percentage of replicate trees in which the associated taxa clustered together in the bootstrap test (1000 replicates) are shown below the branches (*2*). The evolutionary distances were computed using the Kimura 2-parameter method (*3*) and are in the units of the number of base substitutions per site. This analysis involved 14 nt sequences. Codon positions included were 1st+2nd+3rd+Noncoding. All ambiguous positions were removed for each sequence pair (pairwise deletion option). There were a total of 458 positions in the final dataset. Evolutionary analyses were conducted in MEGA11 (*4,5*)

References

1. Saitou N, Nei M. The neighbor-joining method: a new method for reconstructing phylogenetic trees. Mol Biol Evol. 1987;4:406–25. PubMed

2. Felsenstein J. Confidence limits on phylogenies: An approach using the bootstrap. Evolution. 1985;39:783–91. PubMed https://doi.org/10.1111/j.1558-5646.1985.tb00420.x

3. Kimura M. A simple method for estimating evolutionary rates of base substitutions through comparative studies of nucleotide sequences. J Mol Evol. 1980;16:111–20. PubMed https://doi.org/10.1007/BF01731581

4. Tamura K, Stecher G, Kumar S. MEGA11: Molecular Evolutionary Genetics Analysis Version 11. Mol Biol Evol. 2021;38:3022–7. PubMed https://doi.org/10.1093/molbev/msab120

5. Stecher G, Tamura K, Kumar S. Molecular Evolutionary Genetics Analysis (MEGA) for macOS. Mol Biol Evol. 2020;37:1237–9. PubMed https://doi.org/10.1093/molbev/msz312
